# Outpatient Antipsychotic Use and Severe COVID-19: Avoiding the Impact of Age in a Real-World Data Study

**DOI:** 10.1093/ijnp/pyae020

**Published:** 2024-04-11

**Authors:** Samuel Pintos-Rodríguez, Irene Visos-Varela, Almudena Rodríguez-Fernández, Maruxa Zapata-Cachafeiro, María Piñeiro-Lamas, María Teresa Herdeiro, Rosa María García-Álvarez, Adolfo Figueiras, Ángel Salgado-Barreira

**Affiliations:** Department of Public Health, University of Santiago de Compostela, Santiago de Compostela, Spain; Department of Public Health, University of Santiago de Compostela, Santiago de Compostela, Spain; Health Research Institute of Santiago de Compostela (IDIS), Santiago de Compostela, Spain; Consortium for Biomedical Research in Epidemiology and Public Health (CIBER Epidemiology and Public Health - CIBERESP), Madrid, Spain; Department of Public Health, University of Santiago de Compostela, Santiago de Compostela, Spain; Health Research Institute of Santiago de Compostela (IDIS), Santiago de Compostela, Spain; Consortium for Biomedical Research in Epidemiology and Public Health (CIBER Epidemiology and Public Health - CIBERESP), Madrid, Spain; Department of Public Health, University of Santiago de Compostela, Santiago de Compostela, Spain; Health Research Institute of Santiago de Compostela (IDIS), Santiago de Compostela, Spain; Department of Medical Sciences, iBiMED-Institute of Biomedicine, University of Aveiro, Aveiro, Portugal; Santiago de Compostela Health Area, Galician Health Service (Servizo Galego de Saúde - SERGAS), Santiago de Compostela, Spain; Department of Public Health, University of Santiago de Compostela, Santiago de Compostela, Spain; Health Research Institute of Santiago de Compostela (IDIS), Santiago de Compostela, Spain; Consortium for Biomedical Research in Epidemiology and Public Health (CIBER Epidemiology and Public Health - CIBERESP), Madrid, Spain; Department of Public Health, University of Santiago de Compostela, Santiago de Compostela, Spain; Health Research Institute of Santiago de Compostela (IDIS), Santiago de Compostela, Spain; Consortium for Biomedical Research in Epidemiology and Public Health (CIBER Epidemiology and Public Health - CIBERESP), Madrid, Spain; Department of Public Health, University of Santiago de Compostela, Santiago de Compostela, Spain

**Keywords:** Antipsychotics, COVID-19, hospitalization, real-world data, age

## Abstract

**Background:**

The association between use of antipsychotics and COVID-19 outcomes is inconsistent, which may be linked to use of these drugs in age-related diseases. Furthermore, there is little evidence regarding their effect in the nongeriatric population. We aim to assess the association between antipsychotic use and risk of disease progression and hospitalization due to COVID-19 among the general population, stratifying by age.

**Methods:**

We conducted a population-based, multiple case-control study to assess risk of hospitalization, with cases being patients with a PCR(+) test who required hospitalization and controls being individuals without a PCR(+) test; and risk of progression to hospitalization, with cases being the same as those used in the hospitalization substudy and controls being nonhospitalized PCR(+) patients. We calculated adjusted odds-ratios (aOR) and 95% confidence intervals (CI), both overall and stratified by age.

**Results:**

Antipsychotic treatment in patients younger than 65 years was not associated with a higher risk of hospitalization due to COVID-19 (aOR 0.94 [95%CI = 0.69–1.27]) and disease progression among PCR(+) patients (aOR 0.96 [95%CI = 0.70–1.33]). For patients aged 65 years or older, however, there was a significant, increased risk of hospitalization (aOR 1.58 [95% CI = 1.38–1.80]) and disease progression (aOR 1.31 [95% CI = 1.12–1.55]).

**Conclusions:**

The results of our large-scale real-world data study suggest that antipsychotic use is not associated with a greater risk of hospitalization due to COVID-19 and progression to hospitalization among patients younger than 65 years. The effect found in the group aged 65 years or older might be associated with off-label use of antipsychotics.

**Significance Statement**

Our large population-based real-world data study assess the possible effect of antipsychotic medication, stratifying by age group, on COVID-19 outcomes of hospitalization and progression. In contrast to previous studies, our results do not show an increased risk of progression and hospitalization in participants aged younger than 65 years taking antipsychotic medication. For the group aged 65 years or older, the increases in risk found could be due to the confounding effect of off-label use in conditions (i.e., end-of-life care, dementia, etc.) associated with COVID-19 poor prognosis. These results indicate that the use of antipsychotics in patients aged younger than 65 years with COVID-19 is safe, so that discontinuation or modification of antipsychotic treatment in this group of patients would not be justified.

## INTRODUCTION

Antipsychotics (AP) are used in a great variety of psychiatric conditions, though their use has also been widely extended to conditions falling outside the drug prospectus (Højlund et al., 2021; Wang et al., 2021). Indeed, the proportion of off-label AP use represents an important part of total prescriptions and can even exceed that of prescriptions under indication (Hálfdánarson et al., 2017; Højlund et al., 2021; Wang et al., 2021); between 40.0% and 75.0% of AP prescribed in the adult population are for off-label indications, and this figure may increase to 86.3% in institutionalized elderly population (Kamble et al., 2010; Carton et al., 2015). In the case of persons of advanced age, it has been suggested that off-label AP use may be related to frailty (Boland and Dratcu, 2021), a condition defined as “diminished strength, endurance and reduced psychological function that increases an individual’s vulnerability for developing increased dependency and risk of hospitalization or death.” (Hewitt et al., 2020; Sablerolles et al., 2021; Jiang et al., 2023). Prevalence of frailty at a European level has been estimated at around 18% for persons aged 65 years or older (O´Caoimh et al., 2021), with systematic reviews having reported an increase in COVID-19 mortality associated with a greater degree of frailty (Kastora et al., 2021; Pranata et al., 2021).

Studies that have evaluated the association between AP and COVID-19 outcomes display inconsistent results. While on the one hand, AP use has been associated with a higher risk of susceptibility (Cascini et al., 2022), severe illness (Cheng et al., 2023; Fico et al., 2023), and COVID-19 mortality (Poblador-Plou et al., 2020; Reilev et al., 2020; Bliek-Bueno et al., 2021; Harrison et al., 2021; Vai et al., 2021; Cascini et al., 2022; Chen et al., 2023; Cheng et al., 2023; Fico et al., 2023; Secnik et al., 2023), on the other hand, a reduction in (Liberman et al., 2022; Nemani et al., 2022) or absence of effect on susceptibility (Nemani et al., 2022) and mortality (Diez-Quevedo et al., 2021; Nemani et al., 2021; Visser et al., 2023) has also been reported. As possible sources of these inconsistencies, the following have been suggested: (1) most of the studies have been conducted with institutionalized patients with some type of psychiatric disease and/or advanced age, and there are only a few population-based studies (Boland and Dratcu, 2021); (2) a great proportion of these studies do not evaluate the effect of AP by reference to active ingredient or generation (Boland and Dratcu, 2021); (3) off-label prescription of AP in patients of advanced age with dementia or delusions is an indicator of frailty, a condition that is, in turn, a potential risk factor for mortality or other severe COVID-19 outcomes (Hewitt et al., 2020; Kastora et al., 2021; Pranata et al., 2021; Sablerolles et al., 2021); and lastly, (4) many studies that have evaluated the association between AP use and COVID-19 have relied on secondary databases (Reilev et al., 2020; Bliek-Bueno et al., 2021; Diez-Quevedo et al., 2021; Nemani et al., 2021, 2022; Cascini et al., 2022; Liberman et al., 2022; Chen et al., 2023; Secnik et al., 2023; Visser et al., 2023), which pose difficulties in terms of measuring degree of frailty (Reilev et al., 2020; Bliek-Bueno et al., 2021; Cascini et al., 2022; Liberman et al., 2022; Chen et al., 2023). This is because some of the items used in the frailty index calculation are rarely collected on these databases, such as drowsiness, dyspnea, memory problems, tremors, sleep disturbances, need for mobility aids, assistance with dressing, feeding, or grooming (Clegg et al., 2016; Rebora et al., 2023). By secondary databases, we refer to datasets that were not originally collected with the purpose of addressing the relationship under study; these data can be obtained from various sources, such as electronic medical records, health insurance records, or other sources of information previously collected for clinical or administrative purposes (Schneeweiss and Avorn, 2005; Terris et al., 2007).

We therefore felt that it could be of interest to conduct a population-based study in which the effect of AP medication was evaluated by pharmacological group and generation, thereby minimizing the confounding effect of off-label AP use in frail patients. To this end, stratification by age group (<65 vs ≥65 years) would enable us to limit the influence of frailty due to the latter’s low prevalence in patients aged younger than 65 years (O’Caoimh et al., 2018; O´Caoimh et al., 2021). Our study’s main aim was thus to estimate the effect of AP use on risk of hospitalization due to COVID-19 in the sample, both overall and stratified by age; and as a secondary aim, we set out to evaluate the effect of AP on risk of disease progression to stages that might require hospitalization in PCR (+) COVID-19 patients, once again considering the sample overall and stratified by age.

## METHODS

### Study Setting and Background

This study was conducted in Galicia, a region in northwest Spain with approximately 2.7 million inhabitants, where 26.1% of the population is aged 65 years or older and approximately 98% are covered by the Public Health System (PHS). Among the services provided by the PHS are (1) health-related prevention, diagnostic, treatment, and rehabilitation activities; and (2) cost-free medical visits in both primary and hospital care (including access to ambulatory psychiatry visits), though some activities are subject to co-payment (e.g., purchase of certain medications). Because this system is largely funded by taxation, end-users pay only 0% to 60% of the cost of their medication in accordance with their income level. In Spain, as well as in Galicia, medications are mostly dispensed at community pharmacies.

The Galician Health Service (*Servicio Gallego de Salud/SERGAS*), a subsidiary of the national PHS, is tasked with healthcare and data management at a regional level and has access to the electronic medical records of the entire population attended in its catchment area. These electronic medical records record data at both hospital and primary care level, along with data on income level, medical visits, diagnostic tests, treatments (pharmacological or otherwise), and International Classification of Primary Care codes, among other things.

### Study Design and Population

To conduct this study, we used a multiple population-based case-control design. This design is characterized by using all cases (obtained by exhaustive sampling) belonging to a specific, well-identified population (in this case, SERGAS end-users), and comparing the data so obtained against data on participants (controls) who were randomly extracted from the same population as the cases (population-based case controls). This enabled us to obtain an estimate of the prevalence of exposure and the covariates present in the study population.

To achieve this study’s designated objectives, we conducted 2 substudies, in which the definition of case was the same but that of control was different (Table 1).

**Table 1.** Summary of the 2 Case-Control Substudies

Case-control	Progression to hospitalization	Hospitalization
Aim	To evaluate the effect of AP on disease progression to more severe stages that might require hospital admission	To assess the effect of AP on risk of hospitalization for COVID-19
Cases	All participants over the age of 18 years with diagnosis of COVID-19, confirmed by PCR, and admitted to a GHS hospital (n = 2821)	Same cases as those in the progression case-control substudy (n = 2821)
Controls	All patients with diagnosis of COVID-19 confirmed by PCR who did not require hospitalization (n = 26 996)	Participants who did not present a PCR+, matched with cases (n = 52 318) obtained from the general population
Matching	No	Yes^*a*^

Abbreviations: AP, antipsychotics; GHS, Galician Health Service; PCR, polymerase chain reaction.

^*a*^1:20 matched by incidence density, age, sex, primary care service of reference (geographical area), and health professional status.

#### Case-Control Substudy 1: COVID-19 and Risk of Hospitalization

To ascertain the effect of AP use on risk of hospitalization due to COVID-19, cases were defined as all individuals aged 18 years or over, with confirmed diagnosis by PCR (+) test, who required admission to a SERGAS hospital from March 2020 to December 31, 2020. To rule out patients who were admitted for reasons other than COVID-19 infection, we established a maximum difference of 10 days between the date of the PCR (+) test and that of hospitalization. As controls, we randomly selected people belonging to the same population (SERGAS end-users) who, during this period, did not present with a PCR (+) test. To ensure that the risk of exposure to SARS-CoV-2 was as similar as possible, cases and controls were matched (at a ratio of 20 controls per case) by age, sex, and status as health professional or primary-care staff of reference.

#### Case-Control Substudy 2: PCR+ Patient Progression

To estimate the effect of AP use on risk of progression to disease stages that might require hospitalization, cases were defined in the same way as for the hospitalization substudy (individuals aged 18 years or older, with a PCR [+] test and admission to a SERGAS hospital in the period March to December 31, 2020). The controls, in contrast, were all individuals with a diagnosis of COVID-19 confirmed by a PCR (+) test who did not require hospitalization during the same period. The controls were not matched in this case-control substudy, but this would not generate confounding due to the fact that (1) cases and controls were obtained from the same population; (2) they were selected post diagnosis; and (3) the statistical analysis was adjusted for the principal confounding variables.

### Ethical and Legal Aspects

This study was approved by the Galician Clinical Research Ethics Committee (reference 2020-349) and undertaken in accordance not only with prevailing Spanish legislation governing biomedical research and respect for human rights but also with all the requirements stipulated in the Helsinki Declaration. The study protocol was registered in the EU Electronic Register of Post-Authorisation Studies (EUPAS44587) and is available in digital format from https://www.encepp.eu/encepp/viewResource.htm?id=44588.

### Data Source and Collection

All data were obtained from the different databases belonging to the GHS. These databases contain clinical information pertaining to various healthcare levels, including primary care visits, diagnostic tests, surgical interventions, and hospitalizations. The above data were supplemented with further data sourced from healthcare records containing information on the prescription and dispensing of drugs, results of laboratory tests, and the National Health System Hospital discharge registry (Minimum Basic Data Set/ *Conjunto Mínimo Básico de Datos*). All data were extracted semi-automatically by an independent information technology (IT) services company from the Complex Data-Analysis Systems (*Sistemas de Información y Análisis Complejos/SIAC*) used for SERGAS.

### Variable of Exposure and Covariates

The variable of exposure was defined as the prescription and dispensing of any drug belonging to the category of antipsychotics (ATC code N05A) in the 6 months preceding the index date. To rule out cases in which COVID-19 was not the underlying reason for the medical visit or in which the presence of symptoms of the disease might alter exposure to any of these drugs, the index date was set as 10 days before the PCR (+) result: the index date used for the controls of the hospitalization substudy was the same as for the cases with which they were matched. It is important to note here that the use of a specific type of AP is not exclusive, that is, a given patient may use 1 or more AP, whether of the same or a different generation.

We analyzed the effect of AP as a pharmacological group and also by reference to the generation to which a given drug belonged: first-generation antipsychotics (FGA) and second-generation antipsychotics (SGA). These analyses were performed for the total sample and, after stratification by age, for the groups aged younger than 65 years and 65 years or older. The choice of this cut point enabled us to limit the influence of frailty because in persons younger than 65 years, its prevalence is less than 4% (O’Caoimh et al., 2018). Moreover, age over 65 years has already been identified as a risk factor associated with a longer duration of hospital stay (Hewitt et al., 2020) and/or mortality due to COVID-19 (Bonanad et al., 2020; Hewitt et al., 2020; Sablerolles et al., 2021).

As covariates, we collected data on demographic variables, anthropometric variables, and comorbidities (hypertension, diabetes, chronic obstructive pulmonary disease, obesity, ischemic heart disease, cerebrovascular accident, heart failure, atrial fibrillation, chronic renal failure, cancer, asthma), as well as exposure to some type of medication (antihypertensives, nonsteroidal anti-inflammatory drugs, paracetamol, lipid-modifying agents, anticoagulants, antiplatelet agents, glucocorticoids), prescribed and dispensed in the 6 months preceding the index date. To estimate the degree of chronicity of patients, by way of proxy we used the number of medications for chronic conditions prescribed and dispensed in the 6 months preceding the index date, using the proposal from Huber et al. for this purpose (Huber et al., 2013).

### Statistical Analysis

Risk of hospitalization and disease progression in PCR (+) patients were respectively evaluated using multilevel logistic regression. This model was chosen due to the nature and structure of the data used. In addition, these models have a series of advantages over conditional regression models, in that (1) they allow for analysis of matched and unmatched models; (2) they allow for the inclusion of random terms for the control of heterogeneity; and (3) they enable use of information from strata in which all the members have identical exposure values. For construction of the models, random effects were used to assess the effect of the pandemic wave, and nested random effects (3 levels) were used for patients, case-control strata, and health area.

The models were fitted taking into account the main confounding variables, including age, sex, comorbidities, smoking habit, and any drug treatment other than antipsychotics. The results were expressed as adjusted odds ratios (aOR) with their 95% confidence intervals (95% CI). Analyses were performed separately for the sample overall, for patients aged 65 years or older, and for patients younger than 65 years; in each, we evaluated the effect of AP drug use at the level of pharmacological group.

Subsequently, we performed a sensitivity analysis, differentiating by AP generation between FGA and SGA. The active ingredients analyzed for each generation were haloperidol, sulpiride, and levosupliride in the case of FGA; and olanzapine, quetiapine, tiapride, and risperidone in the case of SGA.

Statistical significance was set at .05 and all statistical analyses were performed using the free R Statistical Software environment (version 4.1.0).

## RESULTS

The study population consisted of a total of 82 135 participants. Of these, 29 817 were patients with a PCR (+) test, 2821 of whom required hospitalization. The group of patients without a PCR (+) test comprised the remaining 52 318 participants. Demographic and baseline characteristics of the groups studied are shown in Table 2 for the overall sample and in Table 3 after stratification by age group (<65 vs ≥65 years). As can be seen in Table 3, the presence of comorbidities was far more common in the group aged 65 years or older.

**Table 2.** Demographic and Clinical Characteristics of COVID-19 Cases and Matched Controls (Overall Population)

Characteristic	Hospitalized PCR (+) patients (n = 2821)	Nonhospitalized PCR (+) patients (n = 26 996)	No PCR (+) patients (n = 52 318)
Sex, n (%)			
Male	1457 (51.6)	11 217 (41.6)	26 998 (51.6)
Female	1364 (48.4)	15 779 (58.4)	25 320 (48.4)
Age, median (IQR), y	74 (60–85)	47 (33–63)	73 (60–84)
Health professionals, n (%)	78 (2.8)	1238 (4.6)	1203 (2.3)
Comorbidities, n (%)			
Hypertension	1639 (58.2)	6208 (23)	26 292 (50.3)
Diabetes	782 (27.8)	2519 (9.3)	10 233 (19.6)
COPD	369 (13.1)	759 (2.8)	4305 (8.2)
Obesity	830 (29.5)	3960 (14.7)	10 104 (19.3)
Ischemic heart disease	326 (11.6)	865 (3.2)	4479 (8.6)
Cerebrovascular accident	277 (9.8)	867 (3.2)	3631 (6.9)
Heart failure	430 (15.3)	678 (2.5)	3780 (7.2)
Atrial fibrillation	425 (15.1)	1076 (4)	5405 (10.3)
Chronic renal failure	403 (14.3)	712 (2.6)	4059 (7.8)
Cancer	475 (16.9)	1755 (6.5)	7277 (13.9)
Asthma	267 (9.5)	2170 (8)	3070 (5.9)
Current smoker	737 (26.1)	4180 (15.2)	7842 (15)
Antipsychotics, n (%)			
First-generation AP	174 (6.2)	784 (2.9)	2093 (4.0)
Second-generation AP	252 (8.9)	915 (3.4)	2516 (4.8)

Abbreviations: AP, antipsychotics; COPD, chronic obstructive pulmonary disease; IQR, interquartile range.

**Table 3.** Demographic and Clinical Characteristics of COVID-19 Cases and Matched Controls (Cohorts Aged <65 and ≥65 Years)

	<65 Years			≥65 Years		
Characteristic	Hospitalized PCR (+) patients (n = 908)	Nonhospitalized PCR (+) patients (n = 20 618)	No PCR (+) (n = 17 266)	Hospitalized PCR (+) patients (n = 1913)	Nonhospitalized PCR (+) patients (n = 6378)	Non PCR (+) patients (n = 35 052)
Sex, n (%)						
Male	479 (52.8)	9038 (43.8)	9093 (52.7)	978 (51.1)	2179 (34.2)	17 905 (51.1)
Female	429 (47.2)	11 580 (56.2)	8173 (47.3)	935 (48.9)	4199 (65.8)	17 147 (48.9)
Age, median (IQR), y	53 (44–59)	41 (29–51)	53 (44–59)	81 (74–87)	78 (70–86)	81 (73–87)
Health professionals, n (%)	71 (7.8)	1195 (5.8)	1107 (6.4)	7 (0.4)	43 (0.7)	96 (0.3)
Comorbidities, n (%)						
Hypertension	254 (28)	2138 (10.4)	3207 (18.6)	1385 (72.5)	4070 (63.8)	23 085 (65.9)
Diabetes	139 (15.3)	909 (4.4)	1370 (7.9)	643 (33.6)	1610 (25.2)	8863 (25.3)
COPD	36 (4)	206 (1)	447 (2.6)	333 (17.4)	553 (8.7)	3858 (11)
Obesity	265 (29.2)	2363 (11.5)	2084 (12.1)	565 (29.6)	1597 (25.0)	8020 (22.9)
Ischemic heart disease	30 (3.3)	208 (1)	456 (2.6)	296 (15.5)	657 (10.3)	4023 (11.5)
Cerebrovascular accident	21 (2.3)	167 (0.8)	253 (1.5)	256 (13.4)	700 (11.0)	3378 (9.6)
Heart failure	18 (2)	69 (0.3)	147 (0.9)	412 (21.6)	609 (9.5)	3633 (10.4)
Atrial fibrillation	13 (1.4)	126 (0.6)	191 (1.1)	412 (21.6)	950 (14.9)	5214 (14.9)
Chronic renal failure	24 (2.6)	84 (0.4)	112 (0.6)	379 (19.8)	628 (9.8)	3947 (11.3)
Cancer	65 (7.2)	760 (3.7)	1034 (6)	410 (21.5)	995 (15.6)	6243 (17.8)
Asthma	106 (11.7)	1746 (8.5)	1038 (6)	161 (8.4)	424 (6.6)	2032 (5.8)
Current smoker	294 (32.4)	3393 (16.5)	3899 (22.6)	443 (23.2)	715 (11.2)	3943 (11.2)
Antipsychotics, n (%)						
First-generation AP	32 (3.5)	422 (2.0)	402 (2.3)	142 (7.4)	362 (5.7)	1691 (4.8)
Second-generation AP	28 (3.1)	289 (1.4)	434 (2.5)	224 (11.7)	626 (9.8)	2082 (5.9)

Abbreviations: AP, antipsychotics; COPD, chronic obstructive pulmonary disease; IQR, interquartile range.

### Severe COVID-19 Outcomes: Hospitalization

For the overall sample, we observed that when AP was considered as a single category (without differentiating by generation), there was an increased risk of hospitalization (aOR = 1.44 [95% CI = 1.28–1.62]). Yet, when we stratified by age group, no effect was observed for patients younger than 65 years (aOR = 0.94 [95% CI = .69–1.27]), though it was maintained for those aged 65 years or older (aOR = 1.58 [95% CI = 1.38–1.80]) (see Table 4).

**Table 4.** Association Between Antipsychotic Medication and Risk of Hospitalization and Disease Progression for all Groups

	Overall		<65 Years		≥65 Years	
	aOR (95% CI)	*P*	aOR (95% CI)	*P*	aOR (95% CI)	*P*
Hospitalization						
** **Overall AP	1.44 (1.28 – 1.62)	<.001	0.94 (0.69–1.27)	.687	1.58 (1.38–1.80)	<.001
First-generation AP	1.31 (1.11–1.55)	.001	1.06 (0.72–1.58)	.754	1.39 (1.15–1.67)	<.001
Second-generation AP	1.48 (1.27–1.71)	<.001	0.82 (0.54–1.25)	.364	1.65 (1.41–1.94)	<.001
Disease progression						
Overall AP	1.22 (1.06–1.41)	.006	0.96 (0.70–1.33)	.824	1.31 (1.12–1.55)	.001
First-generation AP	1.26 (1.04–1.53)	.021	0.96 (0.64–1.44)	.833	1.36 (1.09–1.70)	.007
Second-generation AP	1.18 (0.99–1.41)	.071	1.00 (0.63–1.58)	.993	1.25 (1.03–1.51)	.024

Abbreviations: aOR, adjusted odds ratio; AP, antipsychotics.

The results by AP generation were similar to those obtained for AP overall: FGA (aOR = 1.31 [95% CI = 1.11–1.55]; SGA (aOR = 1.48 [95% CI = 1.27–1.71]). When we stratified by age group, this effect disappeared in patients younger than 65 years for both generations: FGA_<65_ (aOR = 1.06 [95% CI = 0.72–1.58]); SGA_<65_ (aOR = 0.82 [95% CI = 0.54–1.25]). Among patients aged 65 years or older, AP use was associated with an increased risk for users of FGA_>65_ (aOR = 1.39 [95% CI = 1.15–1.67]) and SGA_>65_ (aOR = 1.65 [95% CI =1.41–1.94]) alike.

### PCR(+) Patient Progression

The results for the progression substudy displayed a pattern similar to that observed for hospitalization. For the total sample, AP use was associated with an increased risk (aOR = 1.22 [95% CI = 1.06–1.41]). When we stratified by age group, however, a loss of association was observed in the group aged younger than 65 years (aOR = 0.96 [95% CI = 0.70–1.33]), whereas the increased risk was maintained in the group aged 65 years or older (aOR = 1.31 [95% CI = 1.12–1.55]) (the hospitalization results are shown in Table 4).

On analyzing by generation, the results were similar to those obtained for total AP, with an increased risk being observed in both cases, that is, FGA (aOR = 1.26 [95% CI = 1.04 –1.53]) and SGA (aOR = 1.18 [95% CI = 0.99–1.41]). On stratifying by age group, while an absence of effect was observed in the group aged younger than 65 years, FGA_<65_ (aOR = 0.96 95% CI = [0.64–1.44]) and SGA_<65_ (aOR = 1.00 [95% CI = 0.63–1.58]), this increased risk was consolidated in the group aged 65 years or older, FGA_≥65_ (aOR = 1.36 [95% CI = 1.09–1.70]) and SGA_>65_ (aOR = 1.25 [95% CI = 1.03–1.51]).

## DISCUSSION

Previous studies have considered AP use as a potential risk factor for severe COVID-19 outcomes. The results of our large-scale real-world data (RWD) study suggest that AP use is not associated with a greater risk of hospitalization and disease progression in patients younger than 65 years; in those aged 65 years or older, however, a significant increased risk for both outcomes is indeed observed. This difference in effect could be due to off-label use of AP by patients of advanced age with dementia (e.g., Parkinson or Alzheimer disease), depression, delusions, cancer, or as anti-emetics and in end-of life care (Carton et al., 2015; Hálfdánarson et al., 2017; Gerlach et al., 2021; Højlund et al., 2021), conditions that are, in turn, risk factors for severe COVID-19 outcomes (Kozloff et al., 2020; Zheng et al., 2020).

Age is an important determinant of health status and during the pandemic was one of the main risk factors for COVID-19 morbidity-mortality (Bonanad et al., 2020; Williamson et al., 2020; Bliek-Bueno et al., 2021; Baena et al., 2023), with the group aged older than 65 years having a 5-fold higher risk of hospitalization than the reference group aged 18 to 29 years (Baena et al., 2023). This age effect is attributable to social (lack of physical activity, isolation, anxiety due to social stigma, increased difficulty for accessing healthcare services), economic (low income or pension dependent), and biological factors, such as a higher prevalence of dementia and geriatric syndromes, a greater degree of frailty, mobility, or communication problems, and/or lower physiological and functional reserve (Bonanad et al., 2020; Baena et al., 2023), which could therefore act as confounding factors in relation to susceptibility to SARS-CoV-2 infection and severity of the illness (Ruiz de Pellón-Santamaría et al., 2022). Many of these factors are associated with off-label AP use and are thus seldom reflected in the databases of RWD studies, thereby rendering it impossible to adjust for these variables (Reilev et al., 2020; Bliek-Bueno et al., 2021; Cascini et al., 2022; Liberman et al., 2022; Chen et al., 2023).

Antipsychotics are a pharmacological group that is widely prescribed off-label (Carton et al., 2015; Hálfdánarson et al., 2017; Gerlach et al., 2021; Højlund et al., 2021), representing 22% to 86% of total AP prescriptions in persons of advanced age (Carton et al., 2015). Off-label AP prescription ratios vary widely among countries, but some of the factors most strongly associated with off-label use are being institutionalized, manifesting psychotic symptoms, and requiring restraints during hospitalization (Wang et al., 2021). These 3 factors tend to be related with needing sedation more frequently or presenting with more serious comorbidities, which are, in turn, associated with more severe forms and higher COVID-19 mortality (Wang et al., 2021; Cheng et al., 2023).

Most studies that have analyzed the relationship between AP use and the severity of SARS-CoV-2 infection have been conducted within the psychiatric and/or institutionalized population despite the fact that institutionalization is, in itself, a risk factor for presenting with severe COVID-19 outcomes (Canal-Rivero et al., 2021; Diez-Quevedo et al., 2021; Nemani et al., 2021, 2022; Cascini et al., 2022; Liberman et al., 2022; Chen et al., 2023). The particular characteristics of these populations may have an important impact on the results obtained, in view of the fact that (1) congregate living (e.g., nursing homes, hospitals) facilitates propagation of the virus, (2) there is a high prevalence of people with a considerable degree of cognitive decline, and (3) they may present with immunological alterations associated with their comorbidities or treatments (Canal-Rivero et al., 2021). The impossibility of adjusting for some of these confounding variables may result in part of the effects found in other studies on AP use and its link with severe COVID-19 outcomes being attributable to possible confounding by indication (on- or off-label). Moreover, many markers associated with poor COVID-19 prognosis, such as smoking, hypertension, diabetes, and cardiovascular or respiratory diseases (Zheng et al., 2020; Cheng et al., 2023), are highly prevalent among patients with mental diseases (Kozloff et al., 2020; Canal-Rivero et al., 2021).

The absence of effect found in our study for the group aged younger than 65 years could be accounted for by the existence of a mechanism of compensation between (1) the negative effects associated with the severity of underlying disease(s) plus the comorbidities present in AP users, and (2) the potential antiviral effect of certain antipsychotic compounds on SARS CoV-2 and the coronavirus causing MERS-CoV (Middle East respiratory syndrome coronavirus) (Girgis and Lieberman, 2021; Prokopez et al., 2021, 2022; Tendilla-Beltrán et al., 2023). This effect has been observed in vitro, in animal models and in the peripheral blood of patients with schizophrenia (Tendilla-Beltrán and Flores, 2021) and is related with the anti-inflammatory properties, neurotrophic factors, and immunomodulators observed in certain AP compounds (Tendilla-Beltrán and Flores, 2021; Prokopez et al., 2022; Tendilla-Beltrán et al., 2023). The following are the biological mechanisms associated with these properties:

1) Interaction of certain AP with the Spike protein/Angiotensin converting enzyme, related with the ineffectiveness of the virus (Tendilla-Beltrán et al., 2023), and the SIGMA-1 receptor, limiting the replication capacity of the virus (Girgis and Lieberman, 2021; Prokopez et al., 2021, 2022)
2) Inhibition of clathrin-mediated endocytosis (Prokopez et al., 2022; Tendilla-Beltrán et al., 2023), blocking the transport of clathrin and AP2 adaptor complex in the cell membrane, and the ensuing formation of vesicles that would capture and transport the virus to the cell interior (Otręba et al., 2020)
3) Suppression of proinflammatory cytokines such as IL-1A, IL-6, and TNF-α, due to their capacity to modulate the activation of microglia and astrocytes (Tendilla-Beltrán and Flores, 2021; Prokopez et al., 2022), which would attenuate the immune response and inflammatory cascade deriving from SARS-CoV-2 infection.

We feel that our study could well have important clinical and public health implications. Previous studies have found AP to be associated with an increased risk of severe COVID-19 outcomes (Reilev et al., 2020; Bliek-Bueno et al., 2021; Harrison et al., 2021; Cascini et al., 2022; Chen et al., 2023; Cheng et al., 2023; Secnik et al., 2023), which could have led to the partial or complete suspension of the treatment in patients for whom its use is indeed indicated. The results of our study would indicate that, for the group aged younger than 65 years, at least, suspension of AP treatment would not be justified because it might do considerable harm at individual, social, economic, and healthcare levels. The risks associated with untreated or delayed AP treatment are increased psychotic symptoms and cognitive or/functional decline, which consequently diminished the quality and expectancy of life on antipsychotic patients (Fagiolini and Goracci, 2009); this would also have increased direct or indirect costs related to healthcare (Taylor et al., 2023). In case of patients who already were receiving AP treatment, this suspension could induce relapse, with the subsequent burden at a healthcare and economic level in terms of increased frequency of practitioner visits or potential hospitalizations due to a worsening of illness (Fraguas et al., 2008; Taylor et al. 2023).

Our study has a series of advantages. (1) To our knowledge, this is the first study to evaluate the potential effect of AP use on outcomes of disease progression and hospitalization, stratified by age group and AP generation. (2) It included all diagnosed cases of COVID-19 in 2020 in a region of approximately 3 million inhabitants, thereby practically eliminating the influence of selection bias. (3) Its large sample size makes it possible to rule out that the absence of association between AP and severe COVID-19 outcomes in the group younger than 65 years might be due to low statistical power; (4) it was population based, unlike previous studies, which focused on the psychiatric and institutionalized population; and (5) exposure was measured using administrative databases, which reduces the risk of misclassification (Prada-Ramallal et al., 2019).

That said, the study also has a series of limitations. (1) Conclusions cannot be drawn for the group aged 65 years or older because the prevalence of mental disorders or conditions for which off-label AP use is high. (2) The lack of data on all the variables used in the calculation of frailty meant that chronological age was used as an estimator for stratification purposes because there is a good correlation between the two (Mitnitski et al., 2002). (3) Because this was an observational study conducted on the basis of secondary databases, the existence of residual confounding or poorly measured/misclassified variables cannot be ruled out. Also, (4) the fact that antipsychotic treatment is not especially common limited the possibility of conducting subanalyses by active ingredient because there were not enough cases. Finally, although the data used for the study pertain to 2020, when the predominant variants of the virus belonged to class 19B, we nonetheless feel that there is no reason to suppose that the findings observed relate to the dominant variable at that particular point in time.

In conclusion, the results of this large-scale RWD study show no association between AP use and a higher risk of hospitalization due to COVID-19 and/or progression in PCR (+) patients younger than 65 years. Because most of these drugs are administered to persons of advanced age with multiple comorbidities or medical conditions related with more severe forms of COVID-19, one cannot rule out that the associations found in other studies may be due to confounding by indication. Should future studies obtain similar results, this would be an especially relevant finding because it would mean that AP use in the population younger than 65 years is not associated with an increased risk of progression or hospitalization in patients infected with COVID-19, it would therefore not be associated with a higher risk of experiencing COVID-19 complications, and these drugs should be maintained for those cases in which they are indicated.

## Data Availability

Data cannot be shared for ethical/privacy reasons.
